# Efficient double-layer sintering of titanomagnetite concentrate

**DOI:** 10.1038/s41598-022-10405-7

**Published:** 2022-04-15

**Authors:** Liangping Xu, Huibo Liu, De Cheng, Qiang Zhong, Zhiwei Peng, Guanghui Li, Tao Jiang

**Affiliations:** School of Minerals Processing and Bioengineering, Central South University, Changsha, 410083 Hunan China

**Keywords:** Engineering, Materials science

## Abstract

The traditional titanomagnetite sintering process consumes high fuel and produces weak-strength sinter. In this study, double-layer sintering was used to solve above problems. The theoretical analysis and sintering pot test results showed that sintering of feed bed constituted by two different-basicity layers could improve mineralization and permeability. By using the double layer structure of sintering bed and controlling the basicities of lower and upper layer (2.5 and 1.5, respectively), the yield, productivity, and reduction disintegration index (RDI_+3.15_) were 67.32%, 1.65 t m^2^ h^−1^, 49.68% respectively, which were improved 33.12%, 1.74%, and 9.27%, respectively than those obtained by the traditional sintering process. Meanwhile, 1.69 kg t^−1^ of solid fuel consumption and nearly 10% of electricity and gas consumption for sintering were saved. It was demonstrated that using different basicities for upper and lower layer of sintering bed would promote formation of silica-ferrite of calcium and aluminum (SFCA) with simultaneous reduction of perovskite, improving the sinter quality.

## Introduction

Vanadiferous titanomagnetite ores are an important vanadium, titanium, and iron resource^1^. According to the U.S. Geological Survey, it mainly distributed in China, Australia, India, South Africa, and Brazil, of which China accounts for 26.14%^2,3^. These metals are widely applied in metallurgy^4^, electrical engineering^5^, medicine^6–8^, chemical industry^9^, and other fields^10^. Therefore, it is of great significance to utilization of this resource.

The sintering-blast furnace-converter process is one of the most important routes for utilization of titanomagnetite concentrate^11–13^. It was reported that sintering and smelting of titanomagnetite concentrate consume massive energy^14,15^. On the one hand, titanium-bearing ores often have high melting points and require high temperature and long time for sintering ignition, which increases gas consumption^14^. On the other hand, a large amount of heat is wasted by return fines because of its low yield^16,17^. Last but not least, the low reduction disintegration index (RDI_+3.15_) of titanium-containing sinter leads to a high consumption of coke in smelting^18,19^. To solve the above problems, many measures have been proposed. For example, spraying CaCl_2_ could improve the reduction disintegration performance of sinter by strengthening its ability to resist crystal transformation stress from hematite to magnetite in the process of reduction^20,21^. However, chloride would corrode blast furnace and shorten the service life of the furnace. Therefore, it is necessary to explore other better technologies.

Double-layer sintering process proposed in the 1970s was initially proposed to improve the burden structure of blast furnace and reduce the proportion of high-cost pellets with a combination of high basicity sinter and acid sinters^22,23^. Li Jinlong studied the technology with sinter basicity in the range 0.04–3.0 and found it was feasible^24^.

In this study, the double-layer sintering process was used for preparing BF burdens with good quality and high productivity from titanomagnetite concentrate. The permeability of different bed structures was discussed. Subsequently, the effects of bed structure and basicity on the sintering indexes in the sintering process were investigated and compared, with a focus on the effect of TiO_2_ content in the sintering process. This study would provide an effective method to prepare high-quality BF burdens from titanomagnetite resources.

## Experimental

### Raw materials

The raw materials included fine 1, titanomagnetite concentrates A, B and C, quicklime, limestone, and dolomite. Their chemical compositions are shown in Table 1.

**Table 1:** Main chemical composition of raw materials (wt%).

Materials	TFe	SiO_2_	Al_2_O_3_	CaO	MgO	TiO_2_	V_2_O_5_	S	LOI
Fine 1	59.82	5.92	1.23	0.04	0.06	/	/	0.02	5.84
Concentrate A	61.44	2.83	1.71	0.52	0.79	6.60	0.73	0.04	1.30
Concentrate B	63.22	3.46	1.41	2.29	1.54	1.53	0.45	0.02	0.68
Concentrate C	65.48	2.49	1.32	0.91	0.86	2.25	0.50	0.12	0.70
Limestone	0.46	2.77	0.40	51.43	1.05	/	/	0.01	41.35
Dolomite	0.26	3.52	0.02	28.26	21.43	/	/	0.01	44.52
Quicklime	0.83	5.37	0.17	80.47	6.55	/	/	0.01	0.03

*LOI* Loss on ignition.

As shown in Table 1, all the concentrates had high iron grade. To evaluate the effect of titanium oxide, concentrate A with high TiO_2_ content of 6.60% and concentrate B with low TiO_2_ content were used to adjust TiO_2_ content of the mixture of raw materials. The proportion of concentrate C with TiO_2_ content of 2.25% was fixed at 22%. Fine 1 used in the test contained 5.92% SiO_2_ and 59.82% iron, and its proportion was fixed at 12%. Quicklime, limestone, and dolomite were used to control the basicity and MgO contents of sinter product. Coke breeze with fixed carbon of 77.83%, volatile matter of 4.30%, and ash content of 17.87% was used as fuel for sintering.

### Methods

#### Experimental procedure

The sintering tests were completed in a laboratory-scale sintering pot with a dimension of ø180 mm.

In order to obtain two sinter mixtures with different basicity, the raw materials are divided into two parts according to charge calculation. Then, they were individually mixed and granulated for 5 min in a cylinder with dimension of ø600 mm × 1000 mm. Subsequently, the two mixtures were successively charged into the sintering pot to form a double-layer bed structure with entire height of 700 mm. After feeding, ignition was performed at 1100 ± 50 °C with a sucking pressure of 5 kPa at the bottom of sintering pot. After keeping ignition for 2 min, the hot air with temperature above 950 °C was maintained for 1 min to ensure sintering mineralization of the mixture in the upper layer of sintering pot, whilst the sucking pressure was immediately increased to 12 kPa for sintering. The time from the ignition to maximum temperature of flue gas was defined as sintering time. After sintering, the sinter was cooled at − 5 kPa for 3 min, and then crushed, sieved, and collected for analysis.

It is worth noting that the differences between the traditional sintering process (TSP) and double-layer sintering process were that the double-layer sintering process had additional mixing, granulation and charging steps, and the rest steps were completely the same.

### Characterization

The quality of sintering product, namely sinter, was evaluated by several indexes, including the yield, sintering speed (SS), productivity, solid fuel consumption (SFC), tumbler index (TI) and reduction disintegration index (RDI). The yield referred to the mass ratio of sinter > 5 mm and the sintering speed referred to the ratio of the feed height to sintering time. The solid fuel consumption was defined as the amount of coke breeze needed to produce 1 kg of sinter. The TI and RDI values were determined according to the Chinese National Standard Test Methods and Chinese Metallurgical Industry Standard Test Methods.

A scanning electron microscope equipped with energy dispersive spectrometer (SEM–EDS) (FEI, USA) was employed for minerals identification and microstructure analysis. An X-ray diffraction spectrometer (XRD, D/max 2550 PC, Japan Rigaku Co., Ltd) with a Cu anode (wavelength of 1.54056 Å, step scan mode, scanning range of 10°–80°, scanning speed of 5° min^−1^, step length of 0.02°, voltage of 40 kV, and amps of 40 mA) was used to determine the phase compositions of the samples. Besides, an optical microscope (LEICA MDI5000 M, Germany) was applied to characterize the microstructures of the samples. The CaO–TiO_2_–Fe_2_O_3_ ternary phase diagram was calculated by Factsage 7.3.

## Results and discussion

### Permeability analysis of bed structure

Quicklime is of great importance for granulation and mineralization. In this study, it was used to control the basicity of different layers. Moreover, it will also affect granulation. Therefore, it is necessary to discuss the influence of bed structure. To analyze the permeability of different bed structures, the Ergun and Voice equations are given as follows^15,25^.

1 $$ \frac{\Delta P}{H} = 150\frac{{(1 - \varepsilon )^{2} }}{{\varepsilon^{3} }} \cdot \frac{\mu \omega }{{(\varphi d_{p} )^{2} }} + 1.75\frac{1 - \varepsilon }{{\varepsilon^{3} }} \cdot \frac{\rho \cdot \omega }{{\varphi d_{p} }} $$

2 $$ P = \frac{Q}{A}(\frac{H}{\Delta P})^{n} $$

where *ΔP* and *H* are the pressure drop (kg/m^2^) and bed depth (m), respectively, *ε* is the porosity (%), *ρ*, *μ* and *ω* are density (kg/m^3^), viscosity (kg/(m‧s)), and velocity (m/s) of gas, *d*_*p*_ and *φ* are average particle size (m) and shape factor of granule, *P* is the permeability index, *Q* is gas flow (m^3^/min), and *A* is suction area (m^2^). As shown by Eq. (1), the pressure drop is affected by the bed depth, gas properties and mixture properties. Due to use of air, and fixed bed depth, the pressure drop is mainly affected by the properties of mixture. At the same time, the pressure drop determines the permeability index of sintering bed from Eq. (2) when other conditions remain fixed. The smaller the pressure drop was, the better the air permeability was. The distributions of particle size for the mixtures with different basicity are shown in Table 2. The average particle size of high basicity mixture was larger than that of low basicity. Furthermore, the pressure drop of high basicity mixture was smaller. At the basicity of 2.0, the average particle size of the mixture was 4.69 mm, which was smaller than that with basicity of 2.5 and higher than that with basicity of 1.5. Therefore, their permeability obeyed the same trend.

**Table 2:** Particle size distributions of the mixtures with different basicity (%).

Basicity	+ 8 mm	5–8 mm	3–5 mm	1–3 mm	0.5–1 mm	− 0.5 mm	d_ave_/mm
1.5	5.13	25.07	39.89	25.07	4.27	0.57	4.17
2.0	6.59	30.48	48.41	11.09	3.03	0.40	4.69
2.5	13.44	39.68	37.16	7.71	0.00	0.00	5.34

**Figure 1:**
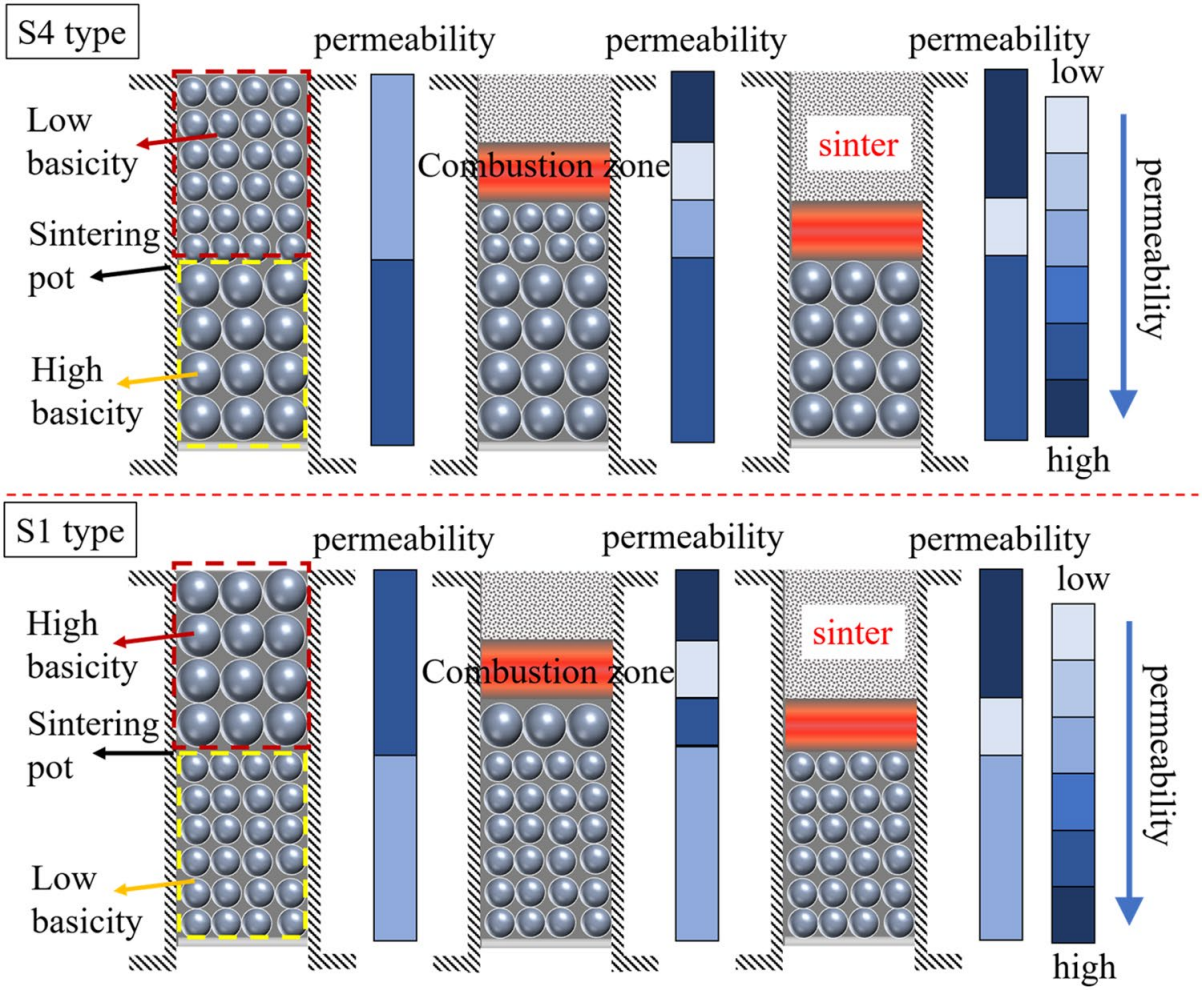
Permeability analysis of different bed structures and sintering states.

To analyze the influence of bed structure on the permeability during the sintering process, the permeability of each zone in different bed structures and sintering states are shown in the Fig. 1.

The bed structure of lower layer with high basicity mixture and upper layer with low basicity mixture was defined as S4 type, while lower layer with low basicity mixture and upper layer with high basicity mixture was defined as S1 type. As illustrated in Fig. 1, the permeability of two types was the same before sintering starting. After ignition, the combustion zone with poor permeability appeared. In the bed structure of S4 type, part of low basicity mixture with poor permeability was transformed into combustion zone with worse permeability, leading to the decrease of permeability for the whole bed. In the bed structure of S1 type, part of high basicity mixture with good permeability was transformed into combustion zone, and the overall permeability of bed decreased substantially. It showed that the difference of permeability for the two types started since ignition, and the permeability of S1 type was lower than that of S4 type. As the combustion zone moved down in the upper layer, the difference increased. When the upper layer sintering was completed, the high basicity lower layer had good permeability in S4 type, while there was a low basicity mixture with poor permeability in S1 type. This difference would exist and affect the sintering speed until the completion of sintering.

As for the traditional sintering process, it had a similar trend. Under the condition of same permeability for the original mixture, the mixture with basicity of 2.0 and low air permeability turned into combustion zone when the upper layer mixture started sintering, which reduced the overall permeability. Its reduction range was larger than that of S4 type, resulting in a poor permeability than that of S4 type. However, the decrease of overall permeability was lower than that of S1 type, which led to better permeability than that of S1 type.

### Sintering pot experiment results

#### Effect of bed structure and basicity

**Figure 2:**
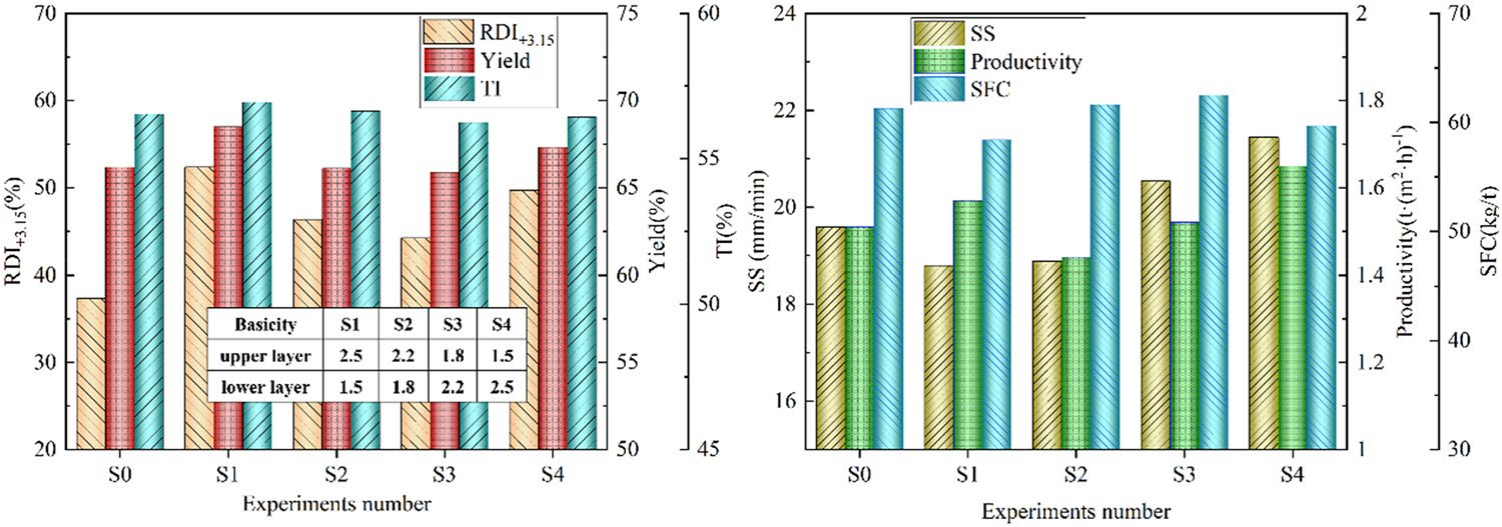
Effect of bed structure and basicity on the double-layer sintering process.

Based on the permeability analysis of bed structure, the corresponding experiments were carried out, and the results are shown in Fig. 2. The TiO_2_ content, comprehensive basicity, and coke breeze dosage was fixed at 2.0%, 2.0 and 3.8%, respectively. Both thicknesses of the upper layer and lower layer were 350 mm. The bed structure is shown in Fig. 2. The S1 and S4 experiments had completely opposite bed structures, so did the S2 and S3 experiments. The S0 was the experiment of TSP.

Figure 2 shows the variations of sintering indexes with the bed structure and basicity. It was found that the sintering indexes of the same basicity and different bed structure were different. The RDI_+3.15_, yield and TI of S2 were higher than those of S3, and the RDI_+3.15_, yield and TI of S1 were higher than those of S4. However, the sintering speed showed the opposite pattern and it indicate that the above permeability analysis is completely correct. Besides, analysis of sinter strength and sintering speed, it could be concluded that the sintering speed affected the crystallization speed of liquid. When the sintering speed was slow, the better crystallization and higher strength would be obtained. However, the effect of sintering speed on productivity is greater than that of yield, which shows that the quality of high alkalinity sintered products is better than that of low alkalinity sintered ores. In addition, the RDI_+3.15_ of sinter improved with the increase of basicity difference between the upper and lower layers. This shows that the RDI_+3.15_ of titanomagnetite sintering under basicity of about 2.0 is the lowest. Increasing or reducing basicity can improve the RDI_+3.15_ efficiently. The lower the alkalinity or the, the higher the RDI_+3.15_. In addition, it shows the trend that the lower or the higher the basicity, the higher the RDI_+3.15_.

Compared with the traditional sintering process with basicity 2.0 and TiO_2_ content of 2.0%, the RDI_+3.15_, yields, and productivity of product from the S1 were increased by 40.24%, 3.51%, and 4.00%, respectively, and a reduction of 2.88 kg_coke_/t_sinter_ SFC was obtained. The RDI_+3.15_, yields, and productivity of product from S4, respectively, achieved 49.68 wt%, 67.32 wt% and 1.65 t m^2^ h^−1^, which were increased by 33.12%, 1.74%, and 9.27%, respectively. Further, close tumbler indexes were achieved and SFC was decreased by 1.69 kg_coke_/t_sinter_. Obviously, the double-layer sintering process could significantly improve productivity and reduce solid fuel consumption compared with the traditional sintering process.

#### Effect of TiO_2_ content on double-layer sintering process

**Figure 3:**
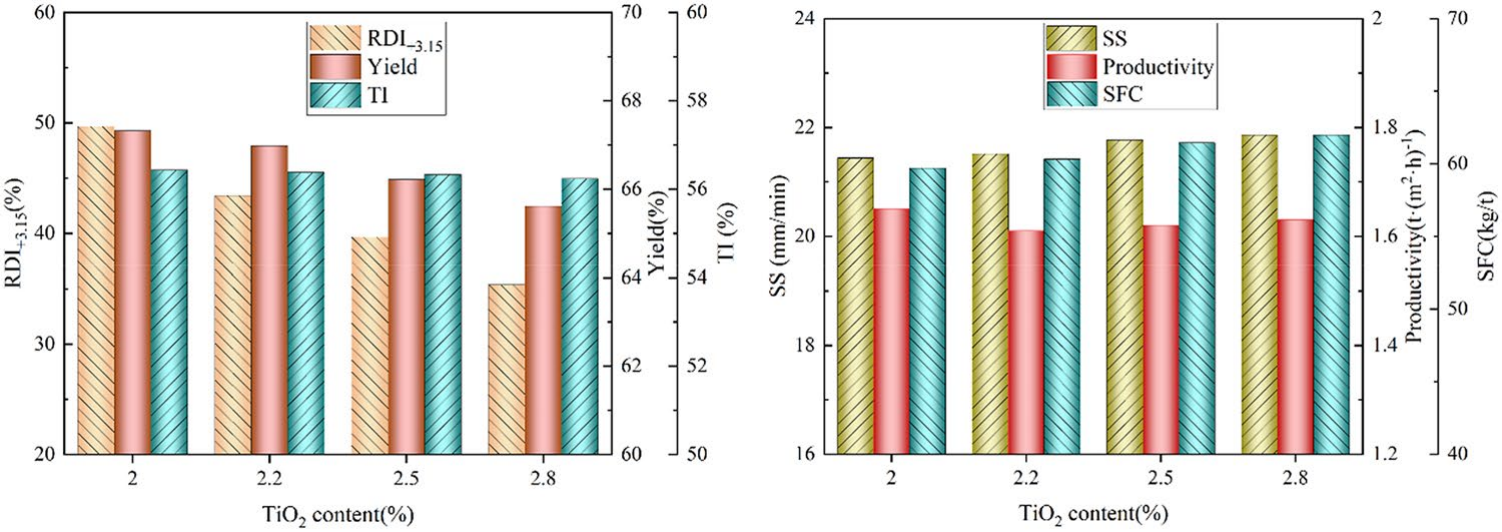
Effect of TiO_2_ content on the double-layer sintering process.

The effect of TiO_2_ content on the double-layer sintering process with S4 type bed structure is shown in Fig. 3. It can be concluded that the RDI_+3.15_ and yield decreased significantly with increasing TiO_2_ content, but the indexes obtained were better than those of traditional sintering process. The TI changed only slightly. The sintering speed and SFC increased slightly, and the productivity decreased to some extent. Under the condition of TiO_2_ content of 2.5%, the yield, RDI_+3.15_, and productivity of double-layer process increased by 2.6%, 27.0% and 8.0%, respectively, compared with traditional sintering, and the SFC reduced by 1.95 kg_coke_/t_sinter_.

### Morphology characterization of different process

**Figure 4:**
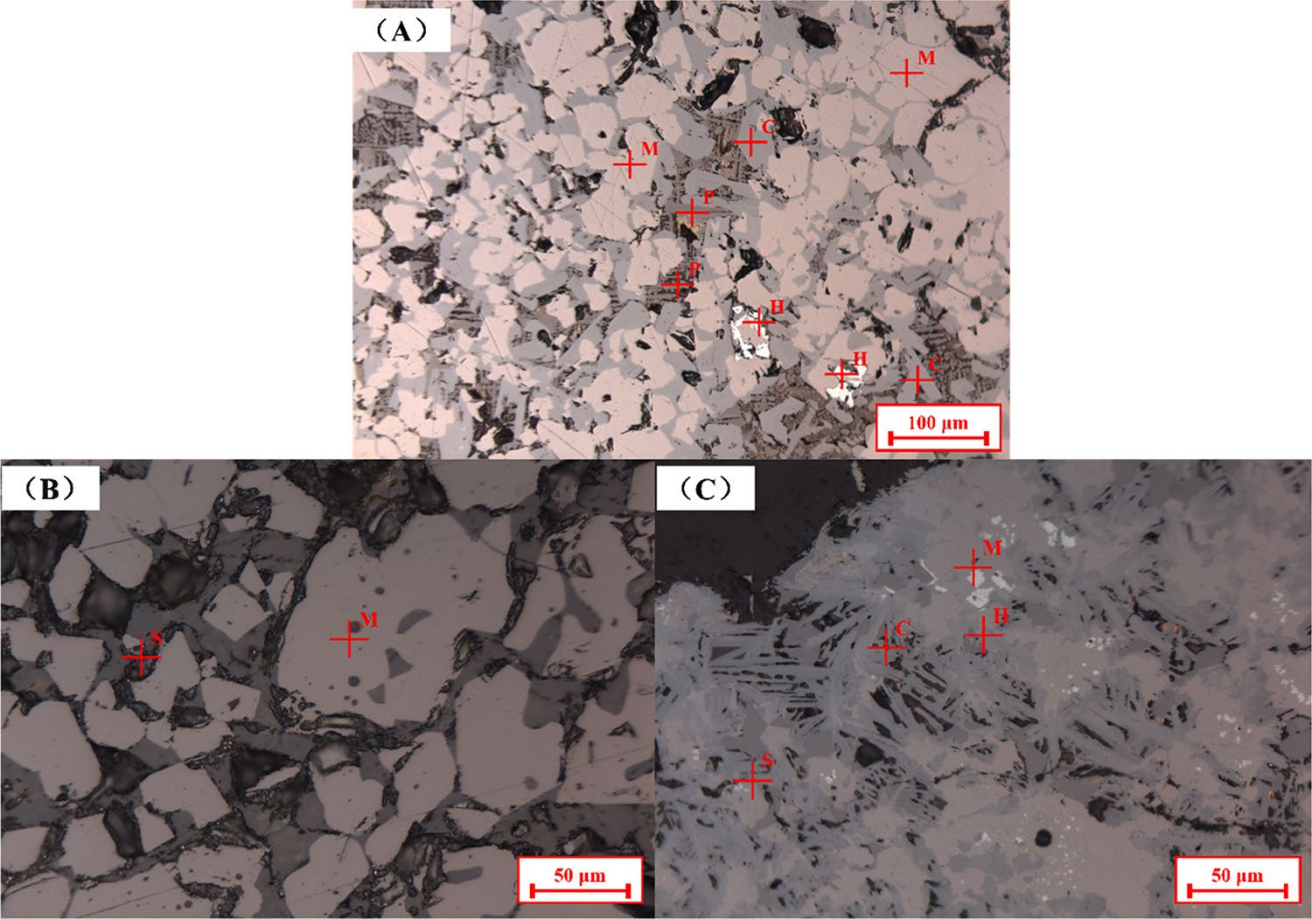
Microstructures of the products from different processes. (**A**) product from TSP with basicity of 2.0 and TiO_2_ content of 2.0%, (**B**) product from upper layer of S4, (**C**) product from lower layer of S4 (H—hematite, M—magnetite, C—SFCA, S—silicate, and P—perovskite).

The microstructures of the products obtained from different sintering processes are shown in Fig. 4. In Fig. 4A, magnetite and calcium ferrite formed interwoven structure, which was related to high strength sinter. Nevertheless, rhombic perovskite crystallized with silicate in sinter, forming a structure that was not good for sinter strength. Such a structure was one of the main reasons for low RDI_+3.15_. The microstructures of the products from the upper layer and lower layer of S4 are shown in Fig. 4B and Fig. 4C. It can be found that the double-layer process was rather different from TSP. The product from the upper layer of S4 with basicity of 1.5 had a relatively simple mineral phase composition, mainly silicates and magnetite. The product from the lower layer of S4 with basicity of 2.5 was mainly composed of SFCA, magnetite, hematite, and silicate. There was no perovskite in Fig. 4B and C. Therefore, it can be explained that the main reason for the good quality of double basicity sintering products is that sufficient calcium oxide forms a high-quality bonding phase in the high basicity sinter, while the low basicity part takes the silicate with general strength as the bonding phase. Unlike traditional sintering, which consumed a lot of CaO combined with TiO_2_, so as to reduce the formation of liquid phase.

**Figure 5:**
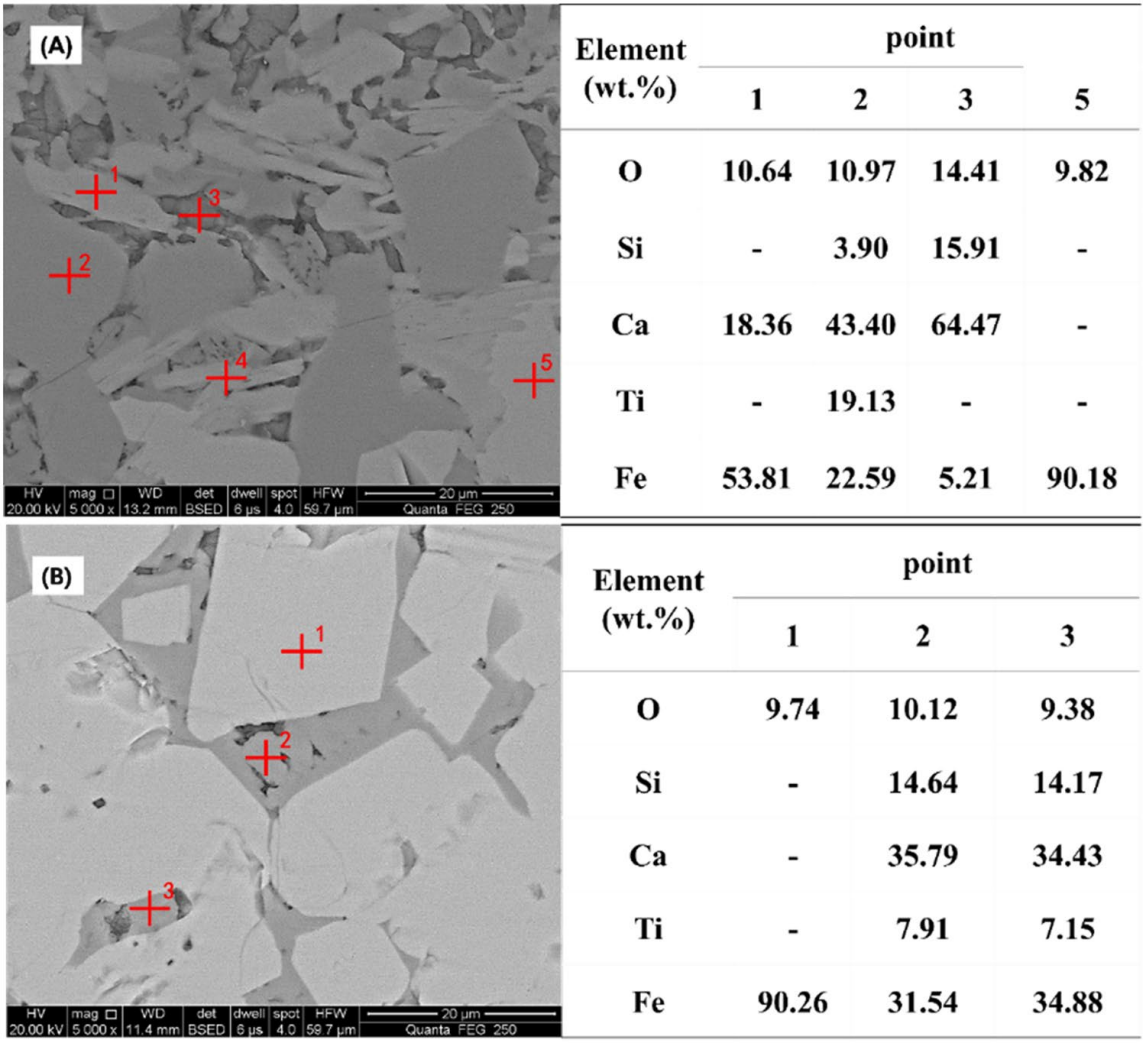
SEM- EDS analysis of sinter produced by the double-layer process. (**A**) product from the lower layer of S4, (**B**) product from the upper layer of S4.

In the double-layer sintering process, the SEM–EDS analysis of the lower layer product from S4 with basicity of 2.5 is shown in Fig. 5A. The main minerals in the product were determined by elemental analysis. SFCA could be observed in the product (point 1) and magnetite was found (point 5). Furthermore, silicate (point 3) was also identified. There were high contents of titanium and calcium and a small amount of silicon in point 2. According to the atomic ratio, perovskite was considered co-dissolved with SFCA in point 2. This explained the disappearance of rhombic perovskite. It is worth noting that there are two different forms of SFCA in the figure. One is dissolved with titanium and silicon, while the other is mainly calcium ferrite. This shows that sufficient CaO can not only complete the reaction of TiO_2_, but also dissolve perovskite with SFCA to reduce its effect. More calcium ferrite will form acicular crystals to ensure the quality of sinter.

The SEM–EDS analysis of the upper layer product from S4 with basicity of 1.5 is shown in Fig. 5B. Compared with Fig. 5A, the microstructure of sinter with basicity of 1.5 was relatively simple. The typical sinter structure with low basicity was formed by the silicate-based binder phase which filled the voids between magnetite particles. Perovskite was fused with silicate, and no rhombic structure was found and deteriorated the sinter strength.

### Mineralization behavior analysis

#### Phase changes in different basicity sintering

**Figure 6:**
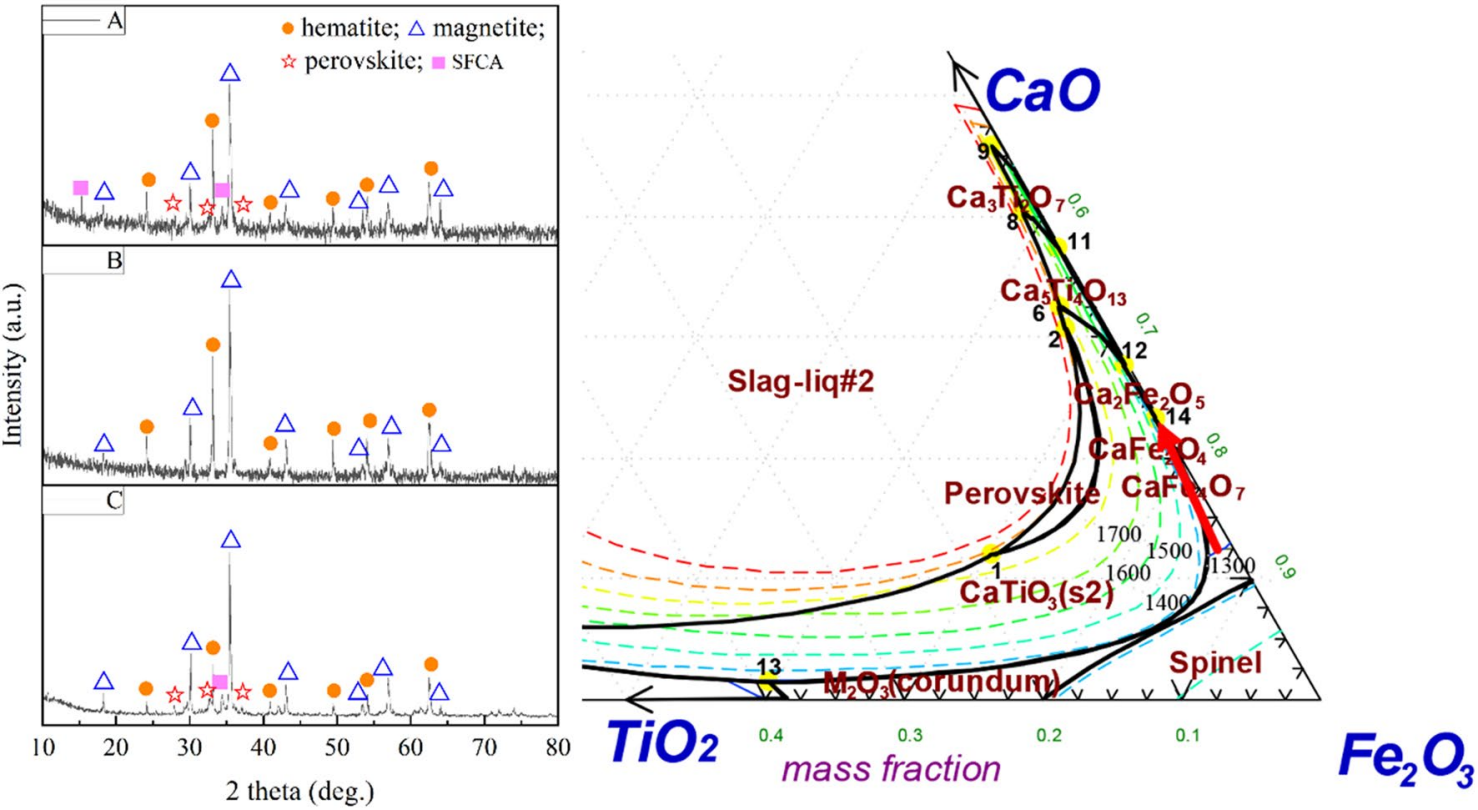
XRD patterns of the sinters obtained with different process and a projection of the liquidus surface of CaO–TiO_2_–Fe_2_O_3_ ternary phase diagram. (**A**—lower layer of S4, **B**—upper layer of S4, and **C**—basicity 2.0 of TSP).

The XRD analyses of the sinters obtained with different basicity and processes are displayed in Fig. 6 left. No peaks of SFCA and perovskite were found in the product with basicity of 1.5 from the upper layer of S4. Compared with the case with basicity of 1.5, the peak number evidently increased in the TSP product with basicity of 2.0 and the product with basicity of 2.5 from the lower layer of S4, indicating the reaction between CaO and TiO_2_ and Fe_2_O_3_. Moreover, the peak intensity of SFCA in the product with basicity of 2.5 from the lower layer of S4 was higher than that of TSP with basicity of 2.0, showing better crystallization. The above results showed that the double-layer process improved RDI_+3.15_ of sinter due to prevention of formation of perovskite by low basicity sintering and reduction of influence of perovskite by high basicity sintering.

#### Liquidus surface analysis with different basicity

In order to further explain the influence of basicity on mineralization during sintering, the ternary phase diagram of CaO–TiO_2_–Fe_2_O_3_ was drawn. The results are shown in Fig. 6. The phase diagram could be used to analyze the liquid phase crystallization process because the sum of Fe_2_O_3_, CaO, TiO_2_ in liquid phase melt was close to 90%. Note that basicity was defined as w(CaO)/w(SiO_2_), but the SiO_2_ content in sinter would not change, generally in the range 4.5–5.2%. Therefore, more attention should be paid to the change of CaO content in the study of basicity.

In the liquidus surface of ternary phase diagram, perovskite would firstly react in a wide range, as indicated by the arrow. The rest of CaO reaction with Fe_2_O_3_ after TiO_2_ was consumed completely. Therefore, in the double-layer sintering process, there was insufficient CaO to react with TiO_2_ to form perovskite when the basicity of mixture was 1.5, which reduced the adverse effect of perovskite on sinter. It can be concluded that at low basicity solid–solid reaction occurs firstly in the mixture to form calcium ferrite with low melting point. With the increase of temperature, SiO_2_ surrounds and melts calcium ferrite, and finally forms glass phase structure^15^. The silicate was the main bonding phase of sinter. On the contrary, at the 2.5 basicity, TiO_2_ was firstly consumed and then a binary system of CaO and Fe_2_O_3_ would form at 1220 °C. Sufficient SFCA would ensure the strength of sinter. The SFCA was the main bonding phase of it.

## Conclusions

In this work, a double-layer sintering process was used to produce blast furnace burdens from titanomagnetite concentrate. The process and traditional sintering process were compared in terms of permeability, sinter indexes, phase composition and microstructure. The ternary phase diagram of CaO–TiO_2_–Fe_2_O_3_ was employed to analyze the effect of CaO content on mineralization. The combination of low basicity in lower layer and high basicity in upper layer of feed bed showed a synergistic effect on improving the quality of titanium-containing sinter and realizing energy conservation. Under the conditions of TiO_2_ content of 2.0 wt%, comprehensive basicity of 2.0, upper layer basicity of 1.5, lower layer basicity of 2.5, and coke breeze dosage of 3.8%, the RDI_+3.15_, yields, productivity, and SFC of sinter were 49.68 wt%, 67.32 wt%, 1.65 t·m^2^·h^−1^, and 59.68 kg t^−1^ s^−1^, respectively.
